# The Glucoamylase Inhibitor Acarbose Has a Diet-Dependent and Reversible Effect on the Murine Gut Microbiome

**DOI:** 10.1128/mSphere.00528-18

**Published:** 2019-02-06

**Authors:** Nielson T. Baxter, Nicholas A. Lesniak, Hamide Sinani, Patrick D. Schloss, Nicole M. Koropatkin

**Affiliations:** a Department of Microbiology and Immunology, University of Michigan Medical School, Ann Arbor, Michigan, USA; UC Davis

**Keywords:** acarbose, gut microbiota, starch

## Abstract

Acarbose is a safe and effective medication for type 2 diabetes that inhibits host glucoamylases to prevent starch digestion in the small intestines and thus decrease postprandial blood glucose levels. This results in an increase in dietary starch in the distal intestine, where it becomes food for the gut bacterial community. Here, we examined the effect of acarbose therapy on the gut community structure in mice fed either a high-starch (HS) or high-fiber diet rich in plant polysaccharides (PP). The fecal microbiota of animals consuming a low dose of acarbose (25 ppm) was not significantly different from that of control animals that did not receive acarbose. However, a high dose of acarbose (400 ppm) with the HS diet resulted in a substantial change to the microbiota structure. Most notably, the HS diet with a high dose of acarbose lead to an expansion of the *Bacteroidaceae* and *Bifidobacteriaceae* and a decrease in the *Verrucomicrobiaceae* (such as Akkermansia muciniphila) and the *Bacteroidales* S24-7. Once acarbose treatment ceased, the community composition quickly reverted to mirror that of the control group, suggesting that acarbose does not irreversibly alter the gut community. The high dose of acarbose in the PP diet resulted in a distinct community structure with increased representation of *Bifidobacteriaceae* and *Lachnospiraceae*. Short-chain fatty acids (SCFAs) measured from stool samples were increased, especially butyrate, as a result of acarbose treatment in both diets. These data demonstrate the potential of acarbose to change the gut community structure and increase beneficial SCFA output in a diet-dependent manner.

**IMPORTANCE** The gut microbial community has a profound influence on host physiology in both health and disease. In diabetic individuals, the gut microbiota can affect the course of disease, and some medications for diabetes, including metformin, seem to elicit some of their benefits via an interaction with the microbiota. Here, we report that acarbose, a glucoamylase inhibitor for type 2 diabetes, changes the murine gut bacterial community structure in a reversible and diet-dependent manner. In both high-starch and high-fiber diet backgrounds, acarbose treatment results in increased short-chain fatty acids, particularly butyrate, as measured in stool samples. As we learn more about how human disease is affected by the intestinal bacterial community, the interplay between medications such as acarbose and the diet will become increasingly important to evaluate.

## Abstract (precis)

The gut microbial community has a profound influence on host physiology in both health and disease. In diabetic individuals, the gut microbiota can affect the course of disease, and some medications for diabetes, including metformin, seem to elicit some of their benefits via an interaction with the microbiota. Here, we report that acarbose, a glucoamylase inhibitor for type 2 diabetes, changes the murine gut bacterial community structure in a reversible and diet-dependent manner. In both high-starch and high-fiber diet backgrounds, acarbose treatment results in increased short-chain fatty acids, particularly butyrate, as measured in stool samples. As we learn more about how human disease is affected by the intestinal bacterial community, the interplay between medications such as acarbose and the diet will become increasingly important to evaluate.

## INTRODUCTION

Diet is an environmental factor that shapes the composition and metabolic output of the bacterial community in the mammalian intestine (1, 2). Metabolites such as short-chain fatty acids (SCFAs) produced by different members of the gut community greatly influence host health (3, 4). SCFAs are the microbial by-products of carbohydrate fermentation, and higher levels generally confer a health benefit, with SCFAs such as propionate, acetate, and butyrate having different tissue fates and host responses (4). Propionate is typically produced by members of the *Bacteroidetes* and is associated with the suppression of cholesterol synthesis but also contributes to gluconeogenesis in the liver (3, 5). Acetate is produced by most members of the *Bacteroidetes*, *Firmicutes*, and *Actinobacteria*, and can locally inhibit virulence genes in enterohemorrhagic Escherichia coli (6) and enter systemic circulation to regulate host energy homeostasis (7). Increased levels of acetate, propionate, and butyrate due to microbial carbohydrate fermentation are implicated in improved host energy balance and the prevention of diet-induced obesity, though acetate and propionate may have a specific role in increasing satiety (3, 4, 8, 9). The SCFA considered to have the most therapeutic potential is butyrate, which is the preferred energy source of colonocytes and has powerful antitumorigenic and anti-inflammatory properties (4, 10). Butyrate appears to strengthen the intestinal epithelial barrier via increased expression of tight junction proteins and has immunosuppressive properties that ameliorate graft versus host disease symptoms in mice after allogeneic bone marrow transplantation (11).

Butyrate is made by a small subset of bacteria, largely within the *Firmicutes* and many of the *Clostridium* cluster XVIa family (12, 13). Enhanced abundance of butyrate-producing organisms is associated with host diets high in dietary fiber, defined as polysaccharides that cannot be accessed by host digestive enzymes (14, 15). One such dietary fiber is resistant starch, which is the portion of starch that is not readily digested by intestinal (gluco)amylases and traverses the distal intestine as food for gut bacteria (16). The digestion of resistant starch is carried out by gut microbes with the unique enzymatic capacity to attack this fiber, and this activity liberates starch oligosaccharides that presumably become food for butyrate-producing species (17–19). However, human volunteer studies using resistant starch to enhance butyrate levels have had mixed success, with some individuals responding to resistant starch consumption via producing more butyrate, and some individuals experiencing no change or reduced butyrate output (18, 20, 21). These changes can largely be attributed to the unique gut microbiota of each individual, which then dictates the response to resistant starch.

Because resistant starch has a generally butyrogenic effect on the gut microbiota, we hypothesized that treatments that increase starch transit to the colon may similarly boost beneficial SCFA output. One treatment for type 2 diabetes and prediabetes is oral administration of host intestinal (gluco)amylase inhibitors, such as acarbose, that competitively inhibit the host (gluco)amylases of the small intestine that are required for starch digestion. Acarbose is a pseudotetrasaccharide that mimics the transition state of the (gluco)amylase hydrolysis reaction and effectively prevents an unsafe postprandial blood glucose increase after starch consumption in individuals with impaired glucose tolerance (22, 23). Acarbose is considered safe due to its local action on intestinal enzymes and minimal absorption into the bloodstream yet tends to be underprescribed in the United States, because treatment requires dosing with each meal and some gastrointestinal discomfort is associated with the start of treatment as starch digestion is shunted to the colon (24). However, these side effects are generally transient and can be avoided by starting at a low dose of acarbose and gradually increasing it over time (24). In addition to diabetes, acarbose has proven to be beneficial in lowering the risk of cardiovascular disease and hypertension (25, 26). Several studies have demonstrated that acarbose treatment in humans generally leads to increases in butyrate as measured in feces or serum, suggesting there is some restructuring of the gut community (27–29). A more recent human volunteer study of prediabetic patients revealed a modest increase in genera associated with short-chain fatty acid production, including *Faecalibacterium*, *Lactobacillus*, and *Prevotella* (30). In another human cohort of type 2 diabetics, acarbose enhanced the abundance of *Bifidobacterium* (31). Beyond the expected variation associated with studies of the human gut community, it may be important to consider how acarbose specifically synergizes with diet, since this medication directly influences host digestion. Because the composition of the microbiota influences the onset and progression of diabetes (32, 33), it is critically important to understand how diabetic medications change the composition and metabolic output of the community. Indeed, recent work with the diabetic medication metformin suggests that at least part of its efficacy is related to its direct effect on the intestinal community composition (34–36).

Here, we investigate the changes in the murine gut community upon feeding a low or high dose of acarbose incorporated into two diet backgrounds. In the first set of experiments with acarbose as part of a high-starch (Western-style) low-fat diet, we observed that a high dose of acarbose was required to dramatically shift the gut microbial community structure and that this effect was directed largely by a significant change in five bacterial operational taxonomic units (OTUs.) Moreover, the effect of acarbose was transient, as the community reverted to the starting (no acarbose) community after the drug was removed from the diet. The specific effects of acarbose on the community were also diet dependent. In the second experiment, we incorporated the high dose of acarbose into a typical high-fiber low-fat rodent chow and observed a dramatic change in the community, distinct from the structure of the high-starch diet plus acarbose. For both diets, an increase in butyrate was observed, but only the high-fiber plus acarbose diet elicited a significant positive change in acetate output. These data demonstrate that acarbose feeding changes the gut community structure in a reversible and diet-dependent manner, which may have implications for how these medications are ideally administered in humans for enhanced therapeutic potential.

## RESULTS

### Acarbose reversibly alters the gut microbiome of mice consuming a high-starch diet.

Prediabetic and diabetic individuals that take acarbose to manage postprandial blood sugar are sometimes started on a low dose of the drug and then switched to a higher therapeutic dose (24). A typical dose in humans is 25 to 50 mg acarbose with each meal at the beginning of treatment, working up to 100 mg with each meal, for a maximum dose of 300 mg/day for a 60-kg adult (37). This progressive dose increase in humans can minimize digestive discomfort associated with the change in starch fermentation in the colon. Previous experiments that administered acarbose to mice incorporated the drug into food at amounts of 1,000 ppm (1 g/1 kg food or 0.1%) (38–40) and 800 ppm (41), although levels as low as 30 ppm improved blood glucose levels in mice with streptozotocin-induced diabetes (42). With this in mind, we used low and high doses of 25 and 400 ppm acarbose, respectively, incorporated into the diet during manufacturing; these were human-equivalent doses of 15 mg and 240 mg per day, respectively, for a 60-kg adult (43). The diet chosen for these experiments was a custom low-fat high-starch (HS) diet in order to mimic a Western-style human diet in which most of the carbohydrate content comes from refined corn starch with little dietary fiber.

**FIG 1:**
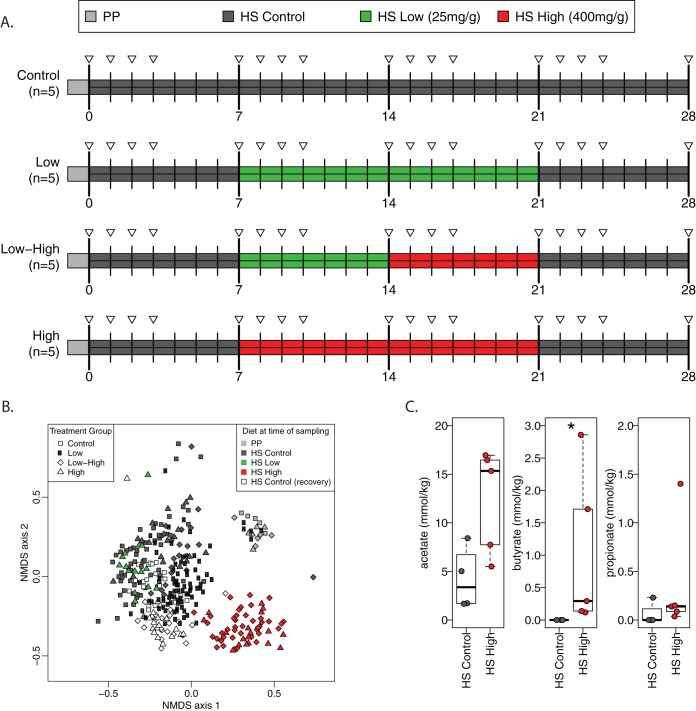
Acarbose reversibly changes the structure of the murine gut community. (A) Experimental setup detailing the four diet groups for the 4-week experiment, color-coded by diet type: PP, plant polysaccharide-rich chow; HS, high starch; control, no acarbose; low, low-dose acarbose; high, high-dose acarbose. The triangles represent when fecal samples were collected and analyzed for 16S rRNA. *N* = 5 mice per group. Note that samples taken on the diet transition day were collected just prior to diet change. (B) NMDS ordination based on Bray-Curtis dissimilarity applied to OTU abundances from fecal samples at time points indicated in A (low versus control diet, ANOSIM *R* = −0.02, *P* = 0.73; high versus control diet, ANOSIM *R* = 0.75, *P* = 0.001). Separate ordinations for each treatment group are compiled in Fig. S1 in the supplemental material. (C) Fecal SCFA concentrations from mice in the HS control and high-dose acarbose groups on day 21. *, *P* < 0.05 by Wilcoxon rank sum test.

To examine the effects of each acarbose dose individually as well as a low to high crossover regime, we used four groups of mice (*n* = 5) between 8 and 12 weeks of age that consumed the HS diet with either zero (control), 25 mg/kg acarbose (low), 400 mg/kg acarbose (high), or a low then high dose of acarbose (low-high) (Fig. 1A). Because many diet changes rapidly alter the murine gut community within 24 to 48 h (44–46), mice were fed the HS control diet for 1 week to allow for adjustment from the standard plant polysaccharide-rich Purina Lab Diet 5001 fed in the UM vivarium. Following 1 week of the HS control diet, the experimental groups consumed the acarbose chow for 2 weeks as outlined above. To determine if the acarbose treatment had a lasting effect on the gut community structure, all groups were switched back to the HS control diet for the last week. The community composition of each group throughout the diet schedule was followed by 16S rRNA gene sequencing of fecal samples on days 1, 2, 3, and 7 of each week of the respective diet.

10.1128/mSphere.00528-18.1 FIG S1 NMDS ordinations by treatment group. Data from Fig. 1B split into separate ordinations. Colors indicate the diet being consumed at the time of sampling. Download FIG S1, PDF file, 0.08 MB. Copyright © 2019 Baxter et al. 2019 Baxter et al. This content is distributed under the terms of the Creative Commons Attribution 4.0 International license.

We used nonmetric multidimensional scaling (NMDS) ordination to visualize the global differences in community structures in the mice between each treatment group over time (Fig. 1B; see also Fig. S1 and S2 in the supplemental material). Overall, the communities clustered more according to the diet consumed at the time of sampling rather than to the diet sequence. Communities from mice consuming the high acarbose dose were significantly different from control mice (analysis of similarity [ANOSIM] *R* = 0.75, *P *= 0.001) and clustered together regardless of whether they went from the control to high acarbose or from low to high acarbose (low-high). Somewhat surprising is that the control and low acarbose groups clustered together, with no significant change in community structure as a result of the 25 mg/kg acarbose (ANOSIM *R* = 0.02, *P *= 0.73). Interestingly, the intestinal communities of mice in the high acarbose or low-high group that went from the high dose to the control diet quickly shifted to cluster with those of the control group, suggesting that acarbose treatment did not irreversibly change the gut community. Beyond the acarbose-mediated change, it is of note that the community shifted dramatically upon switching from the Purina Lab Diet 5001 to the HS diet created from purified protein, starch, and fat sources.

10.1128/mSphere.00528-18.2 FIG S2 NMDS ordination showing the same samples as Fig. 1B, but generated using the Jaccard dissimilarity index. Download FIG S2, PDF file, 0.01 MB. Copyright © 2019 Baxter et al. 2019 Baxter et al. This content is distributed under the terms of the Creative Commons Attribution 4.0 International license.

We expected that acarbose-mediated changes in the community would be apparent as changes in the bacterial metabolic output in the form of short-chain fatty acids, as noted in previous studies (27, 29). We measured short-chain fatty acids in the feces of mice on the HS control and high acarbose diets at day 21 (Fig. 1C). No statistically significant differences were observed in acetate (*P *= 0.063) or propionate (*P *= 0.17) between these groups, but significantly more butyrate (*P *= 0.015) was observed in mice on the high-acarbose diet. Acetate is a fermentation by-product that is common to many anaerobic organisms, regardless of phyla, whereas butyrate is typically associated with *Firmicutes*, specifically, *Clostridiales*, and propionate is typically provided by members of the *Bacteroidetes* (4). The increase in butyrate suggests that acarbose enhances short-chain fatty acid output, which is typically associated with better host health.

**FIG 2:**
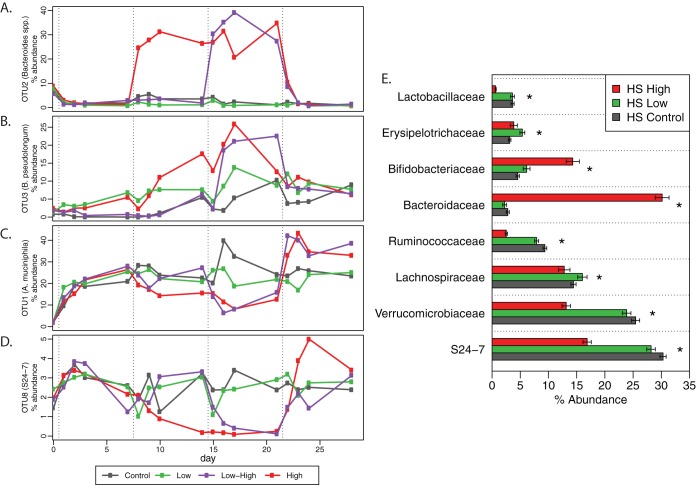
OTU and family-level changes in murine gut as a result of acarbose feeding on a high-starch diet. (A, B) Relative abundances over time of the two OTUs that increased the most in response to acarbose treatment. OTUs that significantly changed in response to acarbose treatment were identified by a Friedman test on each OTU, with Benjamini-Hochberg correction for multiple comparisons. OTU2 and OTU3 had the largest increases in relative abundance among OTUs that significantly changed. (C, D) Relative abundances over time of two OTUs that decreased the most in response to acarbose treatment. OTUs that significantly changed in response to acarbose treatment were identified by repeated measures analysis of variance (ANOVA) on each OTU, with correction for multiple comparisons. OTU1, OTU13, and OTU8 had the largest decreases in relative abundance among OTUs that significantly changed. All OTUs shown had adjusted *P* values of <10^−5^. (E) Relative abundances of the 8 most abundant bacterial families, which accounted for more than 93% of sequences. *, *P* < 0.05 by Wilcoxon rank sum test, high versus control.

To resolve specific changes in the microbiota in response to acarbose, the most responsive OTUs as a result of acarbose treatment in the HS diet were plotted over time (Fig. 2). The OTU with the largest change in abundance was OTU2 (*P *= 1.7 × 10^−5^), which contains sequences that are affiliated with members of the *Bacteroides* (Fig. 2A). OTU2 increased from ∼3% of the total community to ∼30% of the total community in the high-acarbose and low-high groups upon the switch to the high-acarbose diet. Within 24 h of the diet change back to the control HS diet, OTU2 decreased to ∼10% of the total community and then stabilized at a basal level of 2% to 3%, similar to those of the control HS and low acarbose groups. The OTU with second largest increase as a result of the high-acarbose diet is OTU3 (*P *= 1.2 × 10^−5^) that most closely aligned with Bifidobacterium pseudolongum, a known starch degrader (47, 48). The kinetics of the change in this OTU were substantially different from those of OTU2, as OTU3 increased steadily from ∼6% of the community to ∼20% to 25% of the community over the 2-week time period in the high-acarbose group (Fig. 2B). However, this OTU increased somewhat over all groups, including the control group. The low-acarbose group had a somewhat intermediate response in OTU3, from 6% of the community to ∼12% of the total abundance after 2 weeks on the low-acarbose diet. For all groups, including the control, OTU3 stabilized at around 8% to 10% of the community by the end of the 4 weeks, and the withdrawal of acarbose from the experimental groups resulted in a decrease of this community member. The reason for the difference in the kinetics of the OTU3 change between the low-acarbose and low-high groups is unclear, though by the end of the second week when both groups were on the low-acarbose diet, the abundance was similar.

In contrast, two OTUs, OTU1 and OTU8, decreased substantially in response to acarbose dosing (*P *= 2.3 × 10^−5^ and 1.3 × 10^−5^, respectively). OTU1 most closely mapped to Akkermansia muciniphila, a species known for its ability to effectively degrade host mucosal glycans at the exclusion of dietary polysaccharides, including starch (45, 49). OTU1 increased steadily from ∼2% of the community in all groups when the mice were switched from polysaccharide-rich chow and stabilized at around 20% to 25% of the community among all groups at the end of the first week of the HS control diet (Fig. 2C). This change was likely due to the lack of complex dietary fiber (i.e., plant cell wall material) that facilitates the expansion of mucosal glycan degraders as fiber-degrading species decrease (45, 50, 51). The decline in OTU1 was steady over the 2-week course of acarbose treatment in the high-acarbose group, while the low-acarbose group tracked more with the HS control. Interestingly, a rebound effect occurred in OTU1 upon the withdrawal of acarbose in the high-acarbose and low-high groups such that it comprised ∼35% of the community compared to 25% as seen in the low-acarbose group and HS control. A more dramatic decrease in abundance occurred in OTU8 that was classified to the S24-7 family within the *Bacteroidales* (Fig. 2D). This OTU remained at 2% to 3% of the community in the HS control and low-acarbose groups but dropped to the level of detection in all mice consuming a high dose of acarbose. Like OTU1, OTU8 recovered following the withdrawal of acarbose and even exceeded preacarbose levels before it stabilized to the same levels as the control group. The *Bacteroidales* family S24-7 is now well recognized as a typically observed member the intestinal tract of a select set of homeothermic animals and is a core component of the normal mouse microbiota (52, 53). Recent work has suggested the classification of this group as “*Candidatus* Homeothermaceae;” strain differences with this group suggest a flexible metabolism with the ability to digest polysaccharides, including starch, as well as host-derived glycans (54).

Looking more broadly across the eight most abundant bacterial families, the most dramatic changes were the increase in the *Bacteroidaceae* (*P *= 4.4 × 10^−7^) and *Bifidobacteriaceae* (*P *= 4.1 × 10^−7^) upon high acarbose consumption (Fig. 2E), which can be attributed to the expansion of OTU2 and OTU3, respectively. Likewise, the decrease in *Verrucomicrobiaceae* (*P *= 7.3 × 10^−7^) is accounted for by the aforementioned changes in OTU1. Other significant changes that occurred upon high acarbose dosing is a decrease across the *Bacteroidales* S24-7 group (*P* = 2.3 × 10^−5^), *Lactobacillaceae* (*P* = 6.1 × 10^−6^), and *Ruminococcaceae* (*P* = 1.4 × 10^−6^), which occurred across multiple OTUs rather than due to changes in a single taxon. Fifteen OTUs from the *Bacteroidales* S24-7, three from *Lactobacillaceae*, and 25 from *Ruminococcaceae* significantly decreased. In total, 129 OTUs significantly changed in relative abundance response to the high-acarbose treatment, with 31 increasing and 98 decreasing. Together, these data demonstrate a profound change on the bacterial community upon high levels of acarbose consumption, although one that does not persist once treatment is withdrawn.

**FIG 3:**
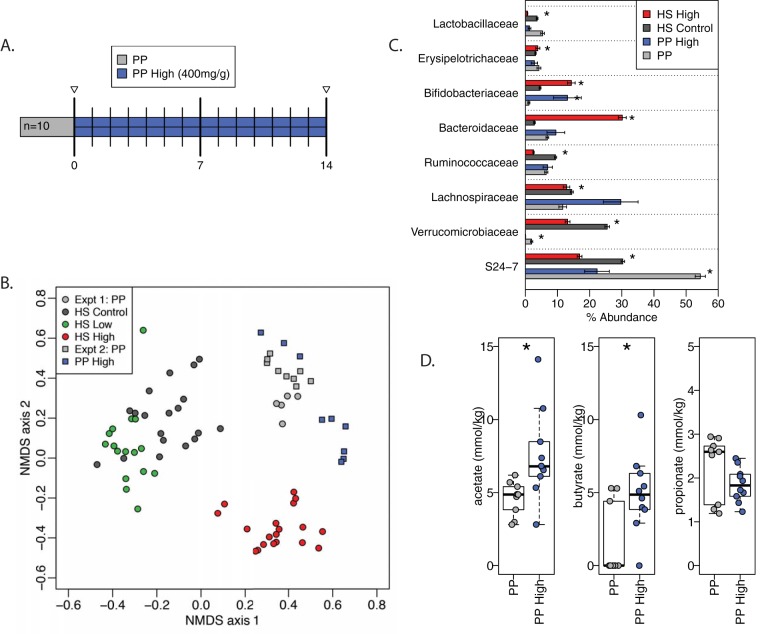
A plant polysaccharide-rich (PP) diet with acarbose changes bacterial taxa differently than a high-starch diet with acarbose. (A) Timeline for PP plus high acarbose diet; *N* = 10 mice. (B) NMDS ordination based on Bray-Curtis dissimilarity applied to OTU abundances within fecal samples from the high-starch (HS) diet experiments (Expt 1) and the plant polysaccharide-rich (PP) diet experiments (Expt 2). Samples from the low-high acarbose group with the HS diet are shown to match the longitudinal design of the PP diet with acarbose. (C) Relative abundances of the 8 most abundant bacterial families, which accounted for more than 93% of sequences. *, *P* < 0.05 by Wilcoxon rank sum test, acarbose versus control/chow. (D) Fecal SCFA concentrations from mice while consuming chow (day 0) or chow with 400 mg/kg acarbose (day 14). *, *P* < 0.05 by Wilcoxon rank sum test.

As noted above, the community structure in fecal samples from mice consuming Purina Lab Diet 5001, a standard rodent chow rich in diverse plant polysaccharides (PP), changed once the animals switched to the HS control diet. This led us to question how acarbose may affect the community dynamics in the context of a different diet. Because the low acarbose dose had little effect on the gut community, we fed a single group of 8- to 12-week-old mice (*n* = 10) a high acarbose (400 mg/kg) dose in the PP-rich Purina Lab Diet,and compared the changes in the gut microbiota before and during acarbose treatment using 16S rRNA gene sequencing as before (Fig. 3A). Plotting these data together over time using NMDS revealed that high acarbose in the context of the PP diet again shifted the community structure, distinct from the communities observed in the HS diet background (Fig. 3B, Fig. S3). Therefore, the effect of acarbose on the community is dependent upon the diet context under which it is administered.

10.1128/mSphere.00528-18.3 FIG S3 NMDS ordination showing the same samples as Fig. 3B, but generated using the Jaccard dissimilarity index. Download FIG S3, PDF file, 0.00 MB. Copyright © 2019 Baxter et al. 2019 Baxter et al. This content is distributed under the terms of the Creative Commons Attribution 4.0 International license.

The relative abundances of bacterial families in the PP diets, both with and without acarbose, were compared to those from our previous experiment with the HS diet background (Fig. 3C). On either diet, high-acarbose treatment resulted in a similar increase in *Bifidobacteriaceae* and decrease in *Lactobacillaceae*, yet many other families responded differently depending on the diet. In the context of the PP diet with acarbose, there was no significant increase in the *Bacteroidaceae* or contraction of the *Ruminococcaceae*, but there was a substantial increase in the *Lachnospiraceae* (though not statistically significant due to the high variability between mice). The PP diet also resulted in lower abundance of the *Verrucomicrobiaceae*, and this family dropped below the limit of detection with acarbose treatment. Finally, the *Bacteroidales* S24-7 group, which was abundant on the HS diet and decreased with acarbose treatment, followed a similar trend with the PP diet, but dominated the community at ∼55% in the PP diet alone and decreased to ∼20% in the PP plus acarbose diet. Thus, at the family level, acarbose selectively changed the community structure in ways that likely affected the metabolic output of the community in the form of short-chain fatty acids and other small molecules that may impact host health. To examine this effect, we measured short-chain fatty acids in the feces of mice at day 0 prior to acarbose feeding on the PP diet and at day 14 after 2 weeks of PP plus acarbose (Fig. 3D). In the PP diet background, high acarbose had statistically significant effects on both acetate (*P* = 0.0057) and butyrate (*P* = 0.033) levels by increasing the detectable amounts of these molecules. Therefore, the ability of acarbose to enhance acetate output by the microbiota was diet dependent, while butyrate was not.

## DISCUSSION

As the composition of the gut microbial community is implicated in the etiology, severity, or progression of human disease, it becomes important to understand how therapeutics alter this community. Medications can have an “off-target” effect in changing the microbiota, and this can be positive or negative depending upon the host background and the magnitude of the effect. For example, proton pump inhibitors are commonly prescribed for the effective treatment of gastric reflux but can alter the gut community and may make individuals more susceptible to intestinal infections (55, 56). Conversely, metformin for the treatment of type 2 diabetes is now thought to elicit better control of blood glucose levels in part because of the changes it drives in the gut bacterial community structure (34–36). As medicine becomes individualized, the interplay between disease, medication, and the microbiota should be considered to maximize therapeutic benefit or prevent unwanted side effects.

Our interest in acarbose originated from the observation that resistant starch, which is starch that is not hydrolyzed and absorbed in the host small intestine, tends to increase butyrate output by the gut microbiota. The therapeutic potential of butyrate is well established and thus, dietary components or treatments that increase this short-chain fatty acid may prevent or halt the progression of colorectal cancer, inflammatory bowel disease, or more systemic inflammatory diseases (10, 15, 57). From our interest in bacterial starch digestion, we questioned how acarbose, a safe and effective glucoamylase inhibitor that is minimally absorbed by the host, might shunt starch to the colon and in turn increase butyrate output. We tested two doses of acarbose in two distinct diet backgrounds, the HS diet which mimics a low-fat Western-style diet comprised of host-accessible processed corn starch and the PP diet replete with plant polysaccharides and natural fiber sources that include resistant starch. The low dose of acarbose, tested only in the HS background, did not result in a significant change in the community structure from that of the control diet lacking acarbose. However, the high-acarbose diet changed the community in the HS diet background with a massive increase in the *Bacteroidaceae* and the *Bifidobacteriaceae*, which in both cases, was largely attributed to increases in single OTUs. There was a nearly concomitant decrease in the abundances of both the *Verrucomicrobiaceae*, mainly, A. muciniphila, and the *Bacteroidales* S24-7 with the high acarbose in the HS background. Acarbose elicited different changes when administered in a PP diet, most notably, a jump in the *Lachnospiraceae* from 10% to 30% of the community with acarbose, and a striking decrease in *Bacteroidales* S24-7. For both diets with acarbose, the abundances of *Bifidobacteriaceae* and *Bacteroidales* S24-7 were quite similar. Acarbose administration enhanced *Bifidobacteriaceae* representation in both cases, whereas it decreased S24-7 in both diets.

Regardless of the precise changes in the gut community, human volunteer studies with acarbose supplementation have reported an increase in serum butyrate levels (27, 29). Therefore, regardless of the precise changes in community structure, there is some conservation of the metabolic footprint observed. We note that the greatest enhancement in butyrate output was observed in the plant polysaccharide diet background. While diets rich in plant fiber tend to have a butyrogenic effect, it is noteworthy that here, the addition of acarbose further enhanced this effect.

From our data, we cannot correlate the changes we see on a community level to what the bacteria are feeding upon in the different diet backgrounds. Certainly, several factors can contribute to these changes. The inhibition of host intestinal glucoamylases by acarbose suggests that there is a substantial increase in starch as an available food source. Starch-targeting enzymes are typically found within glycoside hydrolase family 13 (GH13) and are one of the most well-represented carbohydrate-active enzyme families found within the genomes of many different gut bacteria (58). This is in contrast to the degradation of many complex plant polysaccharides and host mucosal glycans, both of which are more of a specialty food source for select gut bacteria (58, 59). However, because acarbose is minimally absorbed by the host, it transits the distal gut, where it could inhibit starch processing by gut bacteria (60). Therefore, the diet consumed with acarbose greatly influences the possible changes in community structure, as we observed.

Beyond the treatments of diabetes and prediabetes, acarbose has received recent attention as an agent that may extend life span in mice. Acarbose feeding in rodent chow at 1,000 ppm exerts a sex-dependent effect, increasing the median life span of male mice by 22% and of female mice by 5% (39, 61). While the precise mechanism for enhanced longevity is unclear, it is thought to be related to the reduction in postprandial blood glucose elevation or due to lower caloric intake. In these studies, acarbose was administered to animals at vivaria at three different institutions, with notable variability in the effectiveness of acarbose treatment in extending life span and reducing weight gain, despite controlling for mouse breed and diet. Therefore, the changes imposed by acarbose treatment are also affected by the starting gut communities in the animals, which then restrict the potential changes that are observed. Recently, acarbose therapy was evaluated in animal models for the treatment of a variety of other conditions, including cardiovascular disease and cognition (62, 63). As we learn more about how human disease is affected by the intestinal bacterial community, the interplay between medications such as acarbose and the diet will become increasingly important to evaluate.

## MATERIALS AND METHODS

### Animal care.

We used 8- to 12-week-old male C57BL/6 mice obtained from a single breeding colony maintained at the University of Michigan for all of our experiments. These mice were reared under specific-pathogen-free (SPF) conditions within the animal facility at the University of Michigan. All animal-related protocols and experiments were approved by the University Committee on Use and Care of Animals at the University of Michigan and carried out in accordance with the approved guidelines.

### Diets.

All diets were made by Envigo (formerly Harlan Teklad). The high-starch (HS) diet base was AIN-93M, which was comprised of 12.4% protein, 68.3% carbohydrate (46.7% corn starch), and 4.1% fat by weight. The plant polysaccharide-rich standard chow base was the Purina Lab Diet 5001 formulation comprised of 23.5% protein, 40.9% carbohydrate, and 4.5% fat by weight. Acarbose (Sigma-Aldrich) was incorporated into these diets at either 25 mg/kg or 400 mg/kg.

### Feeding regime.

**(i) HS diet experiment.** Four groups (control, high, low, and low-high) of five mice were weaned and consumed a standard Purina Lab Diet 5001 diet for 4 to 8 weeks before switching to the HS control diet. The control group stayed on this diet throughout the 4-week experiment. After 7 days, the low-acarbose group was switched to a 25-mg/kg acarbose HS diet (HS low) for 2 weeks, and the high-acarbose group was switched to 400 mg/kg acarbose (HS high) for 2 weeks. The low-high group consumed 25 mg/kg acarbose for 7 days, then was switched to 400 mg/kg acarbose for another 7 days. The high, low, and low-high groups were all switched back to the control diet at day 21, and mice were humanely sacrificed at day 28. Fecal samples were collected 1, 2, 3, and 7 days after each diet switch to be used for 16S rRNA gene sequencing. Additional stool samples were collected from mice at day 20, flash-frozen in liquid nitrogen, and stored at −80°C for SCFA quantification.

**(ii) PP diet experiment.** One group of ten mice were weaned and consumed a standard Purina Lab Diet 5001 plant polysaccharide diet (PP) for 4 to 8 weeks. A fecal sample was collected from each mouse before switching to a 400 mg/kg acarbose PP diet (PP high) for 14 days. A second fecal sample was collected at the end of the 14 days.

### SCFA quantification.

SCFA analysis was performed by the Thomas Schmidt lab in the Center for Microbial Systems at the University of Michigan. To extract SCFAs, 1 ml of fecal suspension was transferred to a 2-ml 96-well V-bottom collection plate and centrifuged at 4,500 × *g* for 15 min at 4°C. Two hundred microliters of the supernatant fractions was successively filtered through 1.20-, 0.65-, and 0.22-µm 96-well filter plates at 4°C. Filtrates were transferred to 1.5-ml screw-cap vials containing 100-µl inserts in preparation for analysis by high-performance liquid chromatography (HPLC). Quantification of SCFAs was performed using a Shimadzu HPLC system (Shimadzu Scientific Instruments, Columbia, MD) that included an LC-10AD vp pump A, LC-10AD vp pump B, degasser DGU-14A, CBM-20A, autosampler SIL-10AD HT, column heater CTO- 10A(C) vp, UV detector SPD-10A(V) vp, and an Aminex HPX-87H column (Bio-Rad Laboratories, Hercules, CA). We used a mobile phase of 0.01 N H_2_SO_4_ at a total flow rate of 0.6 ml per min with the column oven temperature at 50°C. The sample injection volume was 10 µl and was run for 40 min. Concentrations were calculated using a cocktail of short-chain organic acids standards at concentrations of 20, 10, 5, 2.5, 1, 0.5, 0.25, and 0.1 mM. Concentrations were normalized to the wet weight of fecal material.

### 16S rRNA gene sequencing.

DNA was extracted from mouse fecal pellets using PowerSoil HTP 96-well soil DNA isolation kits (Mo Bio, Carlsbad, CA, USA) and an epMotion 5075 automated pipetting system. The V4 region of the 16S rRNA gene was amplified using custom barcoded primers and sequenced as described previously using 250-bp paired-end reads on an Illumina MiSeq sequencer (64) The 16S rRNA gene sequences were curated using the mothur software package (v1.395), as described previously (64, 65). Briefly, paired-end reads were merged into contigs, screened for quality, aligned to the SILVA 16S rRNA sequence database, and screened for chimeras. Sequences were classified using a naive Bayesian classifier trained against the SILVA 16S rRNA sequence database (66). Curated sequences were clustered into operational taxonomic units (OTUs) using a 97% similarity cutoff with the OptiClust clustering algorithm (67). The number of sequences in each sample was rarefied to 1,960 per sample to minimize the effects of uneven sampling. OTUs that were detected in fewer than 3% of samples were removed to minimize spurious OTUs.

### Statistical analysis.

Coordinates for NMDS ordinations were generated in mothur using thetaYC distances. All other statistical analyses were performed using R (version 3.2.4). Differences in community structures were assessed using ANOSIM with the Bray-Curtis dissimilarity metric. SCFA concentrations were compared using a Wilcoxon rank sum test. OTUs and families that significantly changed in response to the HS high diet were identified by a Friedman test applied to each OTU/family over time blocked according to mouse. The Benjamini-Hochberg method was used to correct *P* values for multiple comparisons. The relative abundances of OTUs or families in response to the PP high diet were compared using paired Wilcoxon rank sum tests with Benjamini-Hochberg correction for multiple comparisons.

### Data availability.

Raw fastq files and metadata are available in NCBI SRA under accession number SRP161616.

## References

[1] Koropatkin NM, Cameron EA, Martens EC 2012 How glycan metabolism shapes the human gut microbiota. Nat Rev Microbiol 10:323–335. doi:10.1038/nrmicro2746. 22491358 PMC4005082

[2] Cockburn D, Koropatkin NM 2016 Polysaccharide degradation by the intestinal microbiota and its influence on human health and disease. J Mol Biol 428:3230–3252. doi:10.1016/j.jmb.2016.06.021. 27393306

[3] Morrison DJ, Preston T 2016 Formation of short chain fatty acids by the gut microbiota and their impact on human metabolism. Gut Microbes 7:189–200. doi:10.1080/19490976.2015.1134082. 26963409 PMC4939913

[4] Koh A, De Vadder F, Kovatcheva-Datchary P, Bäckhed F 2016 From dietary fiber to host physiology: short-chain fatty acids as key bacterial metabolites. Cell 165:1332–1345. doi:10.1016/j.cell.2016.05.041. 27259147

[5] Louis P, Flint HJ 2017 Formation of propionate and butyrate by the human colonic microbiota. Environ Microbiol 19:29–41. doi:10.1111/1462-2920.13589. 27928878

[6] Fukuda S, Toh H, Hase K, Oshima K, Nakanishi Y, Yoshimura K, Tobe T, Clarke JM, Topping DL, Suzuki T, Taylor TD, Itoh K, Kikuchi J, Morita H, Hattori M, Ohno H 2011 Bifidobacteria can protect from enteropathogenic infection through production of acetate. Nature 469:543–547. doi:10.1038/nature09646. 21270894

[7] Kimura I, Ozawa K, Inoue D, Imamura T, Kimura K, Maeda T, Terasawa K, Kashihara D, Hirano K, Tani T, Takahashi T, Miyauchi S, Shioi G, Inoue H, Tsujimoto G 2013 The gut microbiota suppresses insulin-mediated fat accumulation via the short-chain fatty acid receptor GPR43. Nat Commun 4:1829. doi:10.1038/ncomms2852. 23652017 PMC3674247

[8] Lin HV, Frassetto A, Kowalik EJ Jr, Nawrocki AR, Lu MM, Kosinski JR, Hubert JA, Szeto D, Yao X, Forrest G, Marsh DJ 2012 Butyrate and propionate protect against diet-induced obesity and regulate gut hormones via free fatty acid receptor 3-independent mechanisms. PLoS One 7:e35240. doi:10.1371/journal.pone.0035240. 22506074 PMC3323649

[9] Tremaroli V, Backhed F 2012 Functional interactions between the gut microbiota and host metabolism. Nature 489:242–249. doi:10.1038/nature11552. 22972297

[10] Hamer HM, Jonkers D, Venema K, Vanhoutvin S, Troost FJ, Brummer RJ 2008 Review article: the role of butyrate on colonic function. Aliment Pharmacol Ther 27:104–119. doi:10.1111/j.1365-2036.2007.03562.x. 17973645

[11] Mathewson ND, Jenq R, Mathew AV, Koenigsknecht M, Hanash A, Toubai T, Oravecz-Wilson K, Wu SR, Sun Y, Rossi C, Fujiwara H, Byun J, Shono Y, Lindemans C, Calafiore M, Schmidt TC, Honda K, Young VB, Pennathur S, van den Brink M, Reddy P 2016 Gut microbiome-derived metabolites modulate intestinal epithelial cell damage and mitigate graft-versus-host disease. Nat Immunol 17:505–513. doi:10.1038/ni.3400. 26998764 PMC4836986

[12] Barcenilla A, Pryde SE, Martin JC, Duncan SH, Stewart CS, Henderson C, Flint HJ 2000 Phylogenetic relationships of butyrate-producing bacteria from the human gut. Appl Environ Microbiol 66:1654–1661. 10742256 10.1128/aem.66.4.1654-1661.2000 PMC92037

[13] Pryde SE, Duncan SH, Hold GL, Stewart CS, Flint HJ 2002 The microbiology of butyrate formation in the human colon. FEMS Microbiol Lett 217:133–139. doi:10.1111/j.1574-6968.2002.tb11467.x. 12480096

[14] Walker AW, Ince J, Duncan SH, Webster LM, Holtrop G, Ze X, Brown D, Stares MD, Scott P, Bergerat A, Louis P, McIntosh F, Johnstone AM, Lobley GE, Parkhill J, Flint HJ 2011 Dominant and diet-responsive groups of bacteria within the human colonic microbiota. ISME J 5:220–230. doi:10.1038/ismej.2010.118. 20686513 PMC3105703

[15] McIntyre A, Gibson PR, Young GP 1993 Butyrate production from dietary fibre and protection against large bowel cancer in a rat model. Gut 34:386–391. doi:10.1136/gut.34.3.386. 8386131 PMC1374147

[16] Birt DF, Boylston T, Hendrich S, Jane JL, Hollis J, Li L, McClelland J, Moore S, Phillips GJ, Rowling M, Schalinske K, Scott MP, Whitley EM 2013 Resistant starch: promise for improving human health. Adv Nutr 4:587–601. doi:10.3945/an.113.004325. 24228189 PMC3823506

[17] Ze X, Duncan SH, Louis P, Flint HJ 2012 *Ruminococcus bromii* is a keystone species for the degradation of resistant starch in the human colon. ISME J 6:1535–1543. doi:10.1038/ismej.2012.4. 22343308 PMC3400402

[18] Venkataraman A, Sieber JR, Schmidt AW, Waldron C, Theis KR, Schmidt TM 2016 Variable responses of human microbiomes to dietary supplementation with resistant starch. Microbiome 4:33. doi:10.1186/s40168-016-0178-x. 27357127 PMC4928258

[19] Abell GC, Cooke CM, Bennett CN, Conlon MA, McOrist AL 2008 Phylotypes related to *Ruminococcus bromii* are abundant in the large bowel of humans and increase in response to a diet high in resistant starch. FEMS Microbiol Ecol 66:505–515. doi:10.1111/j.1574-6941.2008.00527.x. 18616586

[20] Martinez I, Kim J, Duffy PR, Schlegel VL, Walter J 2010 Resistant starches types 2 and 4 have differential effects on the composition of the fecal microbiota in human subjects. PLoS One 5:e15046. doi:10.1371/journal.pone.0015046. 21151493 PMC2993935

[21] McOrist AL, Miller RB, Bird AR, Keogh JB, Noakes M, Topping DL, Conlon MA 2011 Fecal butyrate levels vary widely among individuals but are usually increased by a diet high in resistant starch. J Nutr 141:883–889. doi:10.3945/jn.110.128504. 21430242

[22] Hillebrand I, Boehme K, Frank G, Fink H, Berchtold P 1979 The effects of the alpha-glucosidase inhibitor BAY g 5421 (acarbose) on meal-stimulated elevations of circulating glucose, insulin, and triglyceride levels in man. Res Exp Med (Berl) 175:81–86. 375342 10.1007/BF01851236

[23] Ibrahim M, Tuomilehto J, Aschner P, Beseler L, Cahn A, Eckel RH, Fischl AH, Guthrie G, Hill JO, Kumwenda M, Leslie RD, Olson DE, Pozzilli P, Weber SL, Umpierrez GE 2018 Global status of diabetes prevention and prospects for action: a consensus statement. Diabetes Metab Res Rev 34:e3201. doi:10.1002/dmrr.3021. 29757486

[24] Hanefeld M 2007 Cardiovascular benefits and safety profile of acarbose therapy in prediabetes and established type 2 diabetes. Cardiovasc Diabetol 6:20. doi:10.1186/1475-2840-6-20. 17697384 PMC2040135

[25] Chiasson JL, Josse RG, Gomis R, Hanefeld M, Karasik A, Laakso M, Group S-NTR 2003 Acarbose treatment and the risk of cardiovascular disease and hypertension in patients with impaired glucose tolerance: the STOP-NIDDM trial. JAMA 290:486–494. doi:10.1001/jama.290.4.486. 12876091

[26] Hanefeld M, Cagatay M, Petrowitsch T, Neuser D, Petzinna D, Rupp M 2004 Acarbose reduces the risk for myocardial infarction in type 2 diabetic patients: meta-analysis of seven long-term studies. Eur Heart J 25:10–16. 14683737 10.1016/s0195-668x(03)00468-8

[27] Wolever TM, Chiasson JL 2000 Acarbose raises serum butyrate in human subjects with impaired glucose tolerance. Br J Nutr 84:57–61. 10961161

[28] Holt PR, Atillasoy E, Lindenbaum J, Ho SB, Lupton JR, McMahon D, Moss SF 1996 Effects of acarbose on fecal nutrients, colonic pH, and short-chain fatty acids and rectal proliferative indices. Metabolism 45:1179–1187. 8781308 10.1016/s0026-0495(96)90020-7

[29] Weaver GA, Tangel CT, Krause JA, Parfitt MM, Jenkins PL, Rader JM, Lewis BA, Miller TL, Wolin MJ 1997 Acarbose enhances human colonic butyrate production. J Nutr 127:717–723. doi:10.1093/jn/127.5.717. 9164992

[30] Zhang X, Fang Z, Zhang C, Xia H, Jie Z, Han X, Chen Y, Ji L 2017 Effects of acarbose on the gut microbiota of prediabetic patients: a randomized, double-blind, controlled crossover trial. Diabetes Ther 8:293–307. doi:10.1007/s13300-017-0226-y. 28130771 PMC5380489

[31] Su B, Liu H, Li J, Sunli Y, Liu B, Liu D, Zhang P, Meng X 2015 Acarbose treatment affects the serum levels of inflammatory cytokines and the gut content of bifidobacteria in Chinese patients with type 2 diabetes mellitus. J Diabetes 7:729–739. doi:10.1111/1753-0407.12232. 25327485

[32] Dunne JL, Triplett EW, Gevers D, Xavier R, Insel R, Danska J, Atkinson MA 2014 The intestinal microbiome in type 1 diabetes. Clin Exp Immunol 177:30–37. doi:10.1111/cei.12321. 24628412 PMC4089152

[33] Mejía-León ME, Petrosino JF, Ajami NJ, Domínguez-Bello MG, de la Barca AMC 2014 Fecal microbiota imbalance in Mexican children with type 1 diabetes. Sci Rep 4:3814. doi:10.1038/srep03814. 24448554 PMC3898044

[34] Forslund K, Hildebrand F, Nielsen T, Falony G, Le Chatelier E, Sunagawa S, Prifti E, Vieira-Silva S, Gudmundsdottir V, Pedersen HK, Arumugam M, Kristiansen K, Voigt AY, Vestergaard H, Hercog R, Costea PI, Kultima JR, Li J, Jorgensen T, Levenez F, Dore J, Meta HITc, Nielsen HB, Brunak S, Raes J, Hansen T, Wang J, Ehrlich SD, Bork P, Pedersen O 2015 Disentangling type 2 diabetes and metformin treatment signatures in the human gut microbiota. Nature 528:262–266. doi:10.1038/nature15766. 26633628 PMC4681099

[35] Wu H, Esteve E, Tremaroli V, Khan MT, Caesar R, Mannerås-Holm L, Ståhlman M, Olsson LM, Serino M, Planas-Fèlix M, Xifra G, Mercader JM, Torrents D, Burcelin R, Ricart W, Perkins R, Fernàndez-Real JM, Bäckhed F 2017 Metformin alters the gut microbiome of individuals with treatment-naive type 2 diabetes, contributing to the therapeutic effects of the drug. Nat Med 23:850–858. doi:10.1038/nm.4345. 28530702

[36] Shin NR, Lee JC, Lee HY, Kim MS, Whon TW, Lee MS, Bae JW 2014 An increase in the *Akkermansia* spp. population induced by metformin treatment improves glucose homeostasis in diet-induced obese mice. Gut 63:727–735. doi:10.1136/gutjnl-2012-303839. 23804561

[37] McIver LA, Tripp J 2018 Acarbose. StatPearls, Treasure Island, FL.

[38] Gibbs VK, Brewer RA, Miyasaki ND, Patki A, Smith DL Jr. 2018 Sex-dependent differences in liver and gut metabolomic profiles with acarbose and calorie restriction in C57BL/6 mice. J Gerontol A Biol Sci Med Sci 73:157–165. doi:10.1093/gerona/glx127. 28651373 PMC5861978

[39] Garratt M, Bower B, Garcia GG, Miller RA 2017 Sex differences in lifespan extension with acarbose and 17-alpha estradiol: gonadal hormones underlie male-specific improvements in glucose tolerance and mTORC2 signaling. Aging Cell 16:1256–1266. doi:10.1111/acel.12656. 28834262 PMC5676051

[40] Petlevski R, Hadzija M, Bajalo JL, Juretic D 2006 Effect of acarbose on alanine aminotransferase and aspartate aminotransferase activities in the liver of control and diabetic CBA mice. Acta Pharm 56:87–93. 16613738

[41] Okada K, Yanagawa T, Warabi E, Yamastu K, Uwayama J, Takeda K, Utsunomiya H, Yoshida H, Shoda J, Ishii T 2009 The alpha-glucosidase inhibitor acarbose prevents obesity and simple steatosis in sequestosome 1/A170/p62 deficient mice. Hepatol Res 39:490–500. doi:10.1111/j.1872-034X.2008.00478.x. 19207582

[42] Casirola DM, Ferraris RP 2006 Alpha-glucosidase inhibitors prevent diet-induced increases in intestinal sugar transport in diabetic mice. Metabolism 55:832–841. doi:10.1016/j.metabol.2006.02.011. 16713445

[43] Reagan-Shaw S, Nihal M, Ahmad N 2008 Dose translation from animal to human studies revisited. FASEB J 22:659–661. doi:10.1096/fj.07-9574LSF. 17942826

[44] David LA, Maurice CF, Carmody RN, Gootenberg DB, Button JE, Wolfe BE, Ling AV, Devlin AS, Varma Y, Fischbach MA, Biddinger SB, Dutton RJ, Turnbaugh PJ 2014 Diet rapidly and reproducibly alters the human gut microbiome. Nature 505:559–563. doi:10.1038/nature12820. 24336217 PMC3957428

[45] Desai MS, Seekatz AM, Koropatkin NM, Kamada N, Hickey CA, Wolter M, Pudlo NA, Kitamoto S, Terrapon N, Muller A, Young VB, Henrissat B, Wilmes P, Stappenbeck TS, Nunez G, Martens EC 2016 A dietary fiber-deprived gut microbiota degrades the colonic mucus barrier and enhances pathogen susceptibility. Cell 167:1339.e21–1353.e21. doi:10.1016/j.cell.2016.10.043. 27863247 PMC5131798

[46] Carmody RN, Gerber GK, Luevano JM Jr, Gatti DM, Somes L, Svenson KL, Turnbaugh PJ 2015 Diet dominates host genotype in shaping the murine gut microbiota. Cell Host Microbe 17:72–84. doi:10.1016/j.chom.2014.11.010. 25532804 PMC4297240

[47] Wang X, Conway PL, Brown IL, Evans AJ 1999 *In vitro* utilization of amylopectin and high-amylose maize (amylomaize) starch granules by human colonic bacteria. Appl Environ Microbiol 65:4848–4854. 10543795 10.1128/aem.65.11.4848-4854.1999 PMC91653

[48] Centanni M, Lawley B, Butts CA, Roy N, Lee JC, Kelly WJ, Tannock GW 2018 *Bifidobacterium pseudolongum* in the ceca of rats fed Hi-Maize starch has characteristics of a keystone species in bifidobacterial blooms. Appl Environ Microbiol 584:e00547-18. doi:10.1128/AEM.00547-18. PMC6052269 29802187

[49] Derrien M, Vaughan EE, Plugge CM, de Vos WM 2004 *Akkermansia muciniphila* gen. nov., sp. nov., a human intestinal mucin-degrading bacterium. Int J Syst Evol Microbiol 54:1469–1476. doi:10.1099/ijs.0.02873-0. 15388697

[50] Pereira FC, Berry D 2017 Microbial nutrient niches in the gut. Environ Microbiol 19:1366–1378. doi:10.1111/1462-2920.13659. 28035742 PMC5412925

[51] Sonnenburg ED, Smits SA, Tikhonov M, Higginbottom SK, Wingreen NS, Sonnenburg JL 2016 Diet-induced extinctions in the gut microbiota compound over generations. Nature 529:212–215. doi:10.1038/nature16504. 26762459 PMC4850918

[52] Seedorf H, Griffin NW, Ridaura VK, Reyes A, Cheng J, Rey FE, Smith MI, Simon GM, Scheffrahn RH, Woebken D, Spormann AM, Van Treuren W, Ursell LK, Pirrung M, Robbins-Pianka A, Cantarel BL, Lombard V, Henrissat B, Knight R, Gordon JI 2014 Bacteria from diverse habitats colonize and compete in the mouse gut. Cell 159:253–266. doi:10.1016/j.cell.2014.09.008. 25284151 PMC4194163

[53] Lagkouvardos I, Pukall R, Abt B, Foesel BU, Meier-Kolthoff JP, Kumar N, Bresciani A, Martinez I, Just S, Ziegler C, Brugiroux S, Garzetti D, Wenning M, Bui TP, Wang J, Hugenholtz F, Plugge CM, Peterson DA, Hornef MW, Baines JF, Smidt H, Walter J, Kristiansen K, Nielsen HB, Haller D, Overmann J, Stecher B, Clavel T 2016 The Mouse Intestinal Bacterial Collection (miBC) provides host-specific insight into cultured diversity and functional potential of the gut microbiota. Nat Microbiol 1:16131. doi:10.1038/nmicrobiol.2016.131. 27670113

[54] Ormerod KL, Wood DL, Lachner N, Gellatly SL, Daly JN, Parsons JD, Dal'Molin CG, Palfreyman RW, Nielsen LK, Cooper MA, Morrison M, Hansbro PM, Hugenholtz P 2016 Genomic characterization of the uncultured *Bacteroidales* family S24-7 inhabiting the guts of homeothermic animals. Microbiome 4:36. doi:10.1186/s40168-016-0181-2. 27388460 PMC4936053

[55] Takagi T, Naito Y, Inoue R, Kashiwagi S, Uchiyama K, Mizushima K, Tsuchiya S, Okayama T, Dohi O, Yoshida N, Kamada K, Ishikawa T, Handa O, Konishi H, Okuda K, Tsujimoto Y, Ohnogi H, Itoh Y 2018 The influence of long-term use of proton pump inhibitors on the gut microbiota: an age-sex-matched case-control study. J Clin Biochem Nutr 62:100–105. doi:10.3164/jcbn.17-78. 29371761 PMC5773837

[56] Tsuda A, Suda W, Morita H, Takanashi K, Takagi A, Koga Y, Hattori M 2015 Influence of proton-pump inhibitors on the luminal microbiota in the gastrointestinal tract. Clin Transl Gastroenterol 6:e89. doi:10.1038/ctg.2015.20. 26065717 PMC4816248

[57] Segain JP, Raingeard de la Blétière D, Bourreille A, Leray V, Gervois N, Rosales C, Ferrier L, Bonnet C, Blottière HM, Galmiche JP 2000 Butyrate inhibits inflammatory responses through NFkappaB inhibition: implications for Crohn's disease. Gut 47:397–403. 10940278 10.1136/gut.47.3.397 PMC1728045

[58] El Kaoutari A, Armougom F, Gordon JI, Raoult D, Henrissat B 2013 The abundance and variety of carbohydrate-active enzymes in the human gut microbiota. Nat Rev Microbiol 11:497–504. doi:10.1038/nrmicro3050. 23748339

[59] Flint HJ, Scott KP, Duncan SH, Louis P, Forano E 2012 Microbial degradation of complex carbohydrates in the gut. Gut Microbes 3:289–306. doi:10.4161/gmic.19897. 22572875 PMC3463488

[60] Santilli AD, Dawson EM, Whitehead KJ, Whitehead DC 2018 Nonmicrobicidal small molecule inhibition of polysaccharide metabolism in human gut microbes: a potential therapeutic avenue. ACS Chem Biol 13:1165–1172. doi:10.1021/acschembio.8b00309. 29660284

[61] Harrison DE, Strong R, Allison DB, Ames BN, Astle CM, Atamna H, Fernandez E, Flurkey K, Javors MA, Nadon NL, Nelson JF, Pletcher S, Simpkins JW, Smith D, Wilkinson JE, Miller RA 2014 Acarbose, 17-alpha-estradiol, and nordihydroguaiaretic acid extend mouse lifespan preferentially in males. Aging Cell 13:273–282. doi:10.1111/acel.12170. 24245565 PMC3954939

[62] Yu MH, Lin MC, Huang CN, Chan KC, Wang CJ 2018 Acarbose inhibits the proliferation and migration of vascular smooth muscle cells via targeting Ras signaling. Vascul Pharmacol 103-105:8–15. doi:10.1016/j.vph.2018.02.001. 29432898

[63] Yan WW, Chen GH, Wang F, Tong JJ, Tao F 2015 Long-term acarbose administration alleviating the impairment of spatial learning and memory in the SAMP8 mice was associated with alleviated reduction of insulin system and acetylated H4K8. Brain Res 1603:22–31. doi:10.1016/j.brainres.2015.01.042. 25645154

[64] Kozich JJ, Westcott SL, Baxter NT, Highlander SK, Schloss PD 2013 Development of a dual-index sequencing strategy and curation pipeline for analyzing amplicon sequence data on the MiSeq Illumina sequencing platform. Appl Environ Microbiol 79:5112–5120. doi:10.1128/AEM.01043-13. 23793624 PMC3753973

[65] Schloss PD, Westcott SL, Ryabin T, Hall JR, Hartmann M, Hollister EB, Lesniewski RA, Oakley BB, Parks DH, Robinson CJ, Sahl JW, Stres B, Thallinger GG, Van Horn DJ, Weber CF 2009 Introducing mothur: open-source, platform-independent, community-supported software for describing and comparing microbial communities. Appl Environ Microbiol 75:7537–7541. doi:10.1128/AEM.01541-09. 19801464 PMC2786419

[66] Pruesse E, Quast C, Knittel K, Fuchs BM, Ludwig W, Peplies J, Glockner FO 2007 SILVA: a comprehensive online resource for quality checked and aligned ribosomal RNA sequence data compatible with ARB. Nucleic Acids Res 35:7188–7196. doi:10.1093/nar/gkm864. 17947321 PMC2175337

[67] Westcott SL, Schloss PD 2017 OptiClust, an improved method for assigning amplicon-based sequence data to operational taxonomic units. mSphere 2:e00073-17. doi:10.1128/mSphereDirect.00073-17. 28289728 PMC5343174

