# Erratum for Baxter et al., “The Glucoamylase Inhibitor Acarbose Has a Diet-Dependent and Reversible Effect on the Murine Gut Microbiome”

**DOI:** 10.1128/mSphere.00347-19

**Published:** 2019-05-22

**Authors:** Nielson T. Baxter, Nicholas A. Lesniak, Hamide Sinani, Patrick D. Schloss, Nicole M. Koropatkin

**Affiliations:** a Department of Microbiology and Immunology, University of Michigan Medical School, Ann Arbor, Michigan, USA

## ERRATUM

Volume 4, issue 1, e00528-18, 2019, https://doi.org/10.1128/mSphere.00528-18. This article was published on 6 February 2019 with the first author’s name misspelled. The byline was updated in the current version, posted on 16 May 2019.

