# Supplementary figures and images for: The Glucoamylase Inhibitor Acarbose Has a Diet-Dependent and Reversible Effect on the Murine Gut Microbiome
Source: mSphere. 2019 Feb 6;4(1):e00528-18. doi: 10.1128/mSphere.00528-18 (PMC6365613; doi:10.1128/mSphere.00528-18)

PP
  HS Control
  HS Low
  HS High
  HS Control (recovery)

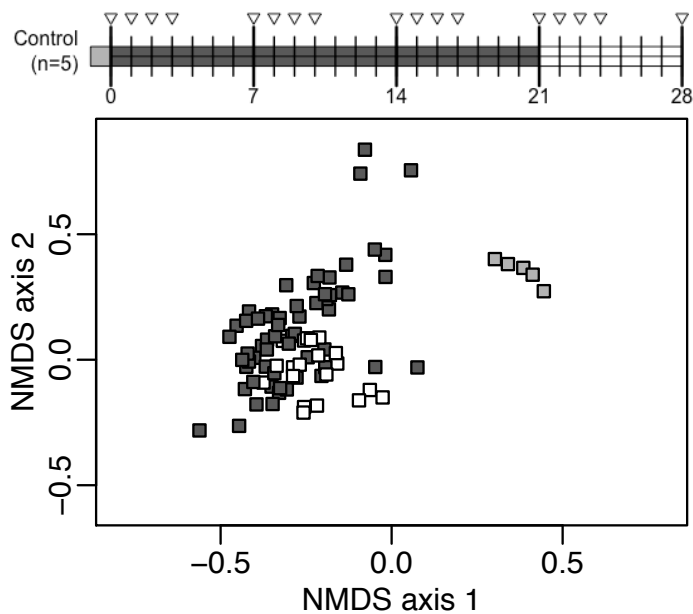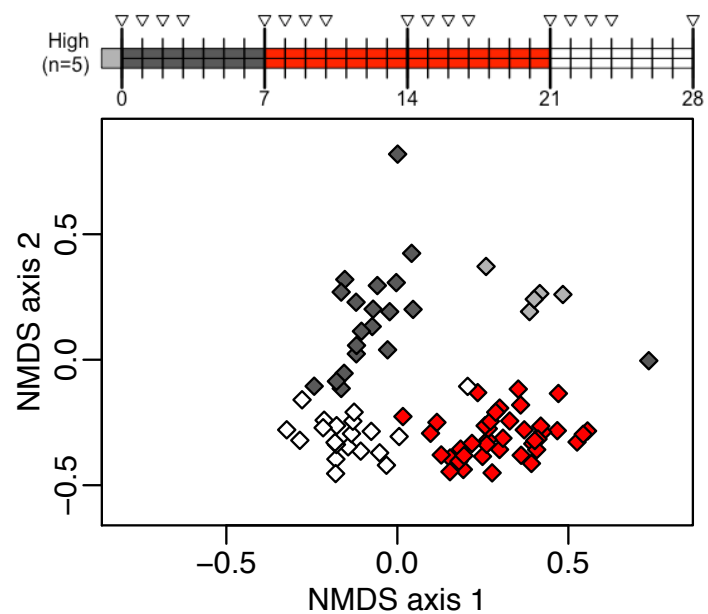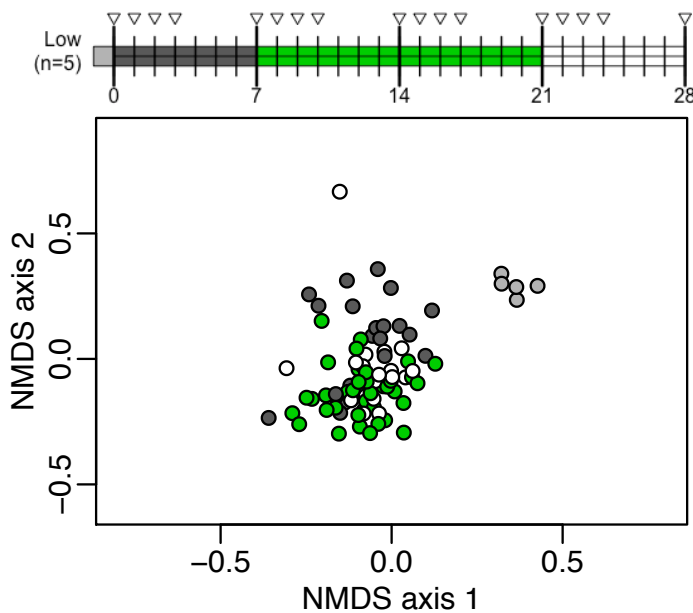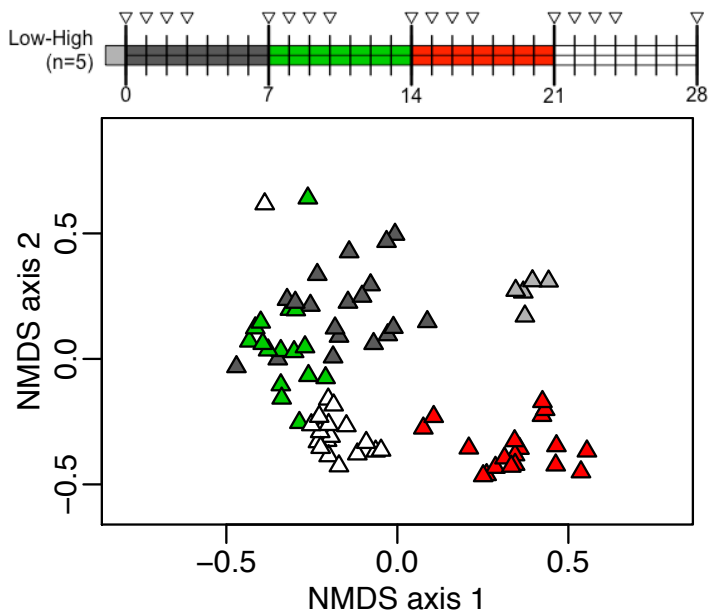

Supplement: FIG S1 [file mSphere.00528-18-sf001.pdf]

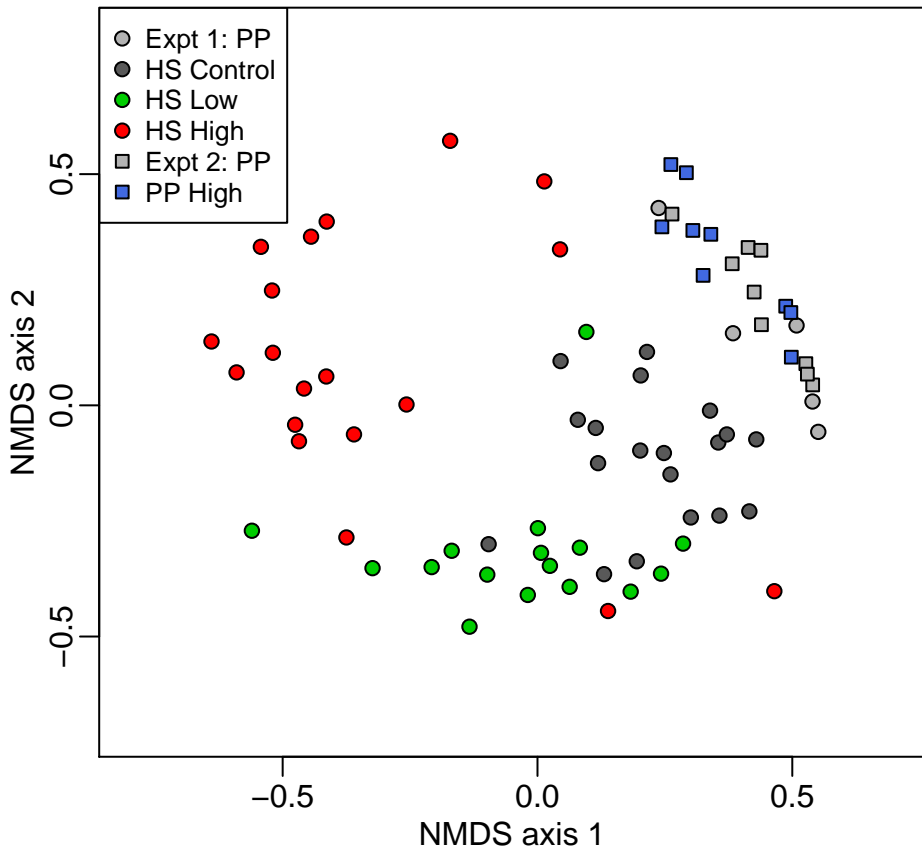

Supplement: FIG S3 [file mSphere.00528-18-sf003.pdf]
