# Supplementary material for: The Glucoamylase Inhibitor Acarbose Has a Diet-Dependent and Reversible Effect on the Murine Gut Microbiome
Source: mSphere. 2019 Feb 6;4(1):e00528-18. doi: 10.1128/mSphere.00528-18 (PMC6365613; doi:10.1128/mSphere.00528-18)
Supplement: FIG S2 [file mSphere.00528-18-sf002.pdf]

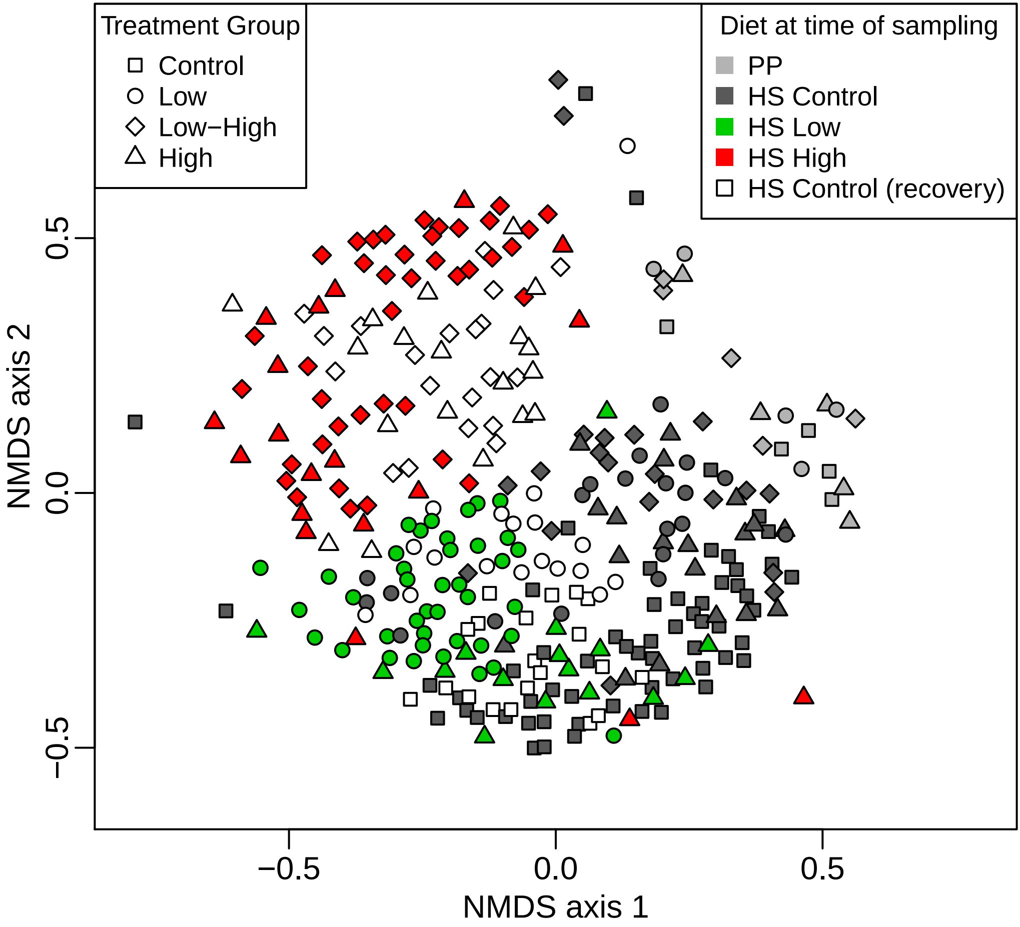
Treatment Group
Control
Low
Low−High
High
Diet at time of sampling
PP
HS Control
HS Low
HS High
HS Control (recovery)
0.5
0.0
−0.5
−0.5
0.0
0.5
NMDS axis 1
NMDS axis 2
